# Meet up‐and‐coming analytical scientists – Mateusz Krzysztof Łącki

**DOI:** 10.1002/ansa.202200045

**Published:** 2022-12-17

**Authors:** Mateusz Krzysztof Łącki

**Affiliations:** ^1^ Institute for Immunology University Medical Center of the Johannes Gutenberg University Mainz Mainz Germany


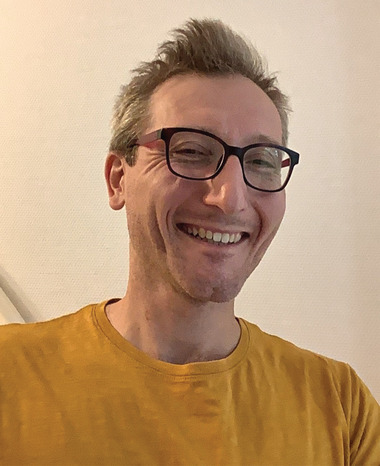
Analytical sciences are among the most dynamically developing fields and have been inherently integrated into many various scientific disciplines. At the same time, Early Career Researchers are among those whose contribution to this dynamic growth cannot be simply overestimated. Hence, in this special issue ‘From one Early Career Researcher to the next’, we are presenting a series of editorials with Q&A from five emerging scientists of different analytical fields, including omics, environmental and data sciences. Importantly, all our guests boast not only scientific excellence and high‐quality research but also the substantial international experience gained during their Ph.D. or postdoctoral training. For this editorial, we are presenting Dr. Mateusz Krzysztof Łącki.

Dr. Mateusz Krzysztof Łącki comes from Warsaw in Poland, where he studied economics at the Warsaw School of Economics, and mathematics at the University of Warsaw (and preferred the latter). After getting master's titles in both fields, he joined the bioinformatics group of Prof. Anna Gambin at the University of Warsaw. There, he completed his Ph.D. in computer science, after which he was awarded the best Ph.D. in bioinformatics prize by the Polish Bioinformatics Society. After that, together with his dear wife, he moved to Mainz and started working at Prof. Tenzer's mass spec core facility at the Johannes Gutenberg University Medical Center.

## What is your original background?

I have a Ph.D. in computer science and both a master's and bachelor's titles in mathematics and computational economics. During the Ph.D., I was introduced to the field of mass spectrometry and collaborated extensively with top–down mass spectrometrists: prof. Frank Sobott, prof. Dirk Valkenborg and Dr. Frederik Lermyte. Back then, we were trying to address problems with a relative lack of software for the analysis of top–down mass spectrometry data in proteomics. In that emerging technique, proteins are fragmented directly in the mass spectrometer instead of being digested by trypsin before being introduced into the instrument. Whole proteins are much larger than peptides, can obtain many more charges during the ionization phase, and their fragmentation in the instrument provides much more observable fragments. Solving this problem basically required writing a whole peptide‐centric analytical pipeline from scratch.

## What is your current research focus?

I am currently helping in the optimization of the construction of the data collection on a timsTOF device. timsTOF mass spectrometers are relatively new instruments used in exploratory proteomics that pair traditional liquid chromatography and mass time‐of‐flight mass spectrometry with ion mobility separation. Why is that advantageous? The biggest problem with applying mass spectrometry in biology is the overall complexity of the sample. This creates a technical problem, as the detector can get overwhelmed by the sheer number of ions. This also creates a data interpretation problem. To overcome these problems, modern mass spectrometry offers quadrupole filtering and fragmentation. Filtering limits what we are currently looking at, and fragmentation shows us the building blocks of peptides. Fortunately, most peptides can be uniquely described by their measured fragments. Organizing the cycles of filtering and fragmentation is referred to as data acquisition. The two most prominent methods are data‐dependent acquisition (DDA) and data‐independent acquisition (DIA). In DDA, an initial complex mass spectrum of peptides is acquired, and a collection of the most abundant signals are selected for further fragmentation. Nothing guarantees, however, that you will fragment the same ions from cycle to cycle. This ultimately leads to missing values in your mass spec reports. On the other hand, in DIA, you filter ions in a predefined order and fragment those. In this way, the measurements are not dependent on a whimsical ion race. The price you pay for more structure though is more interpretational complexity, as you observe at the same time signals coming from multiple peptides. At that moment, the computer takes over and, at least for now, it needs a human to tell it what to do. In addition, what I do is apply modern‐day data analysis algorithms. What we are currently doing is reorganizing the acquisition of data so that it could maximize the number of available hints about the identity of peptides. This includes changes to the instrument done on the producer's side and setting up an entirely new pipeline for the data analysis purpose.

## What is your biggest motivation to work in analytical science?

The best thing is that here it is absolutely essential to apply mathematics and coding to understand what is going on. It is extremely rewarding to see how one can offset the immediate interpretability of measurement against a computer‐driven analysis. We can control where we want more entropy and direct it where we pay less for it (at least timewise: I still get paid). We are using modern analytical instruments and pairing them with machines designed only for computations. Everything seems to have its role here: The ingenuity of the engineers in the making of the instrument goes hand in hand with the abstract thought put into the algorithms we use.

## Of all your research projects, which one was your favourite and why?

My favourite project concerned an algorithm for modelling the isotopic signals of molecules in mass spectrometry. It was done jointly with Dr. Michal Startek from the University of Warsaw. The problem is easy to state: Give me a chemical formula, and I will tell you its mass. Sounds simple, right? However, molecules are composed of atoms, and atoms have isotopes, and each has a different mass due to the extra neutron inside. The isotopes come with different frequencies, so there is a probabilistic vibe to the problem. The task we solved is how to enumerate the most probable masses first; as for bigger molecules measured in, say, top–down mass spectrometry, you would end up with millions of possibilities. The nice thing is that the problem is complex but easy enough to prove that the solution we found is optimal in terms of the number of computations we perform.


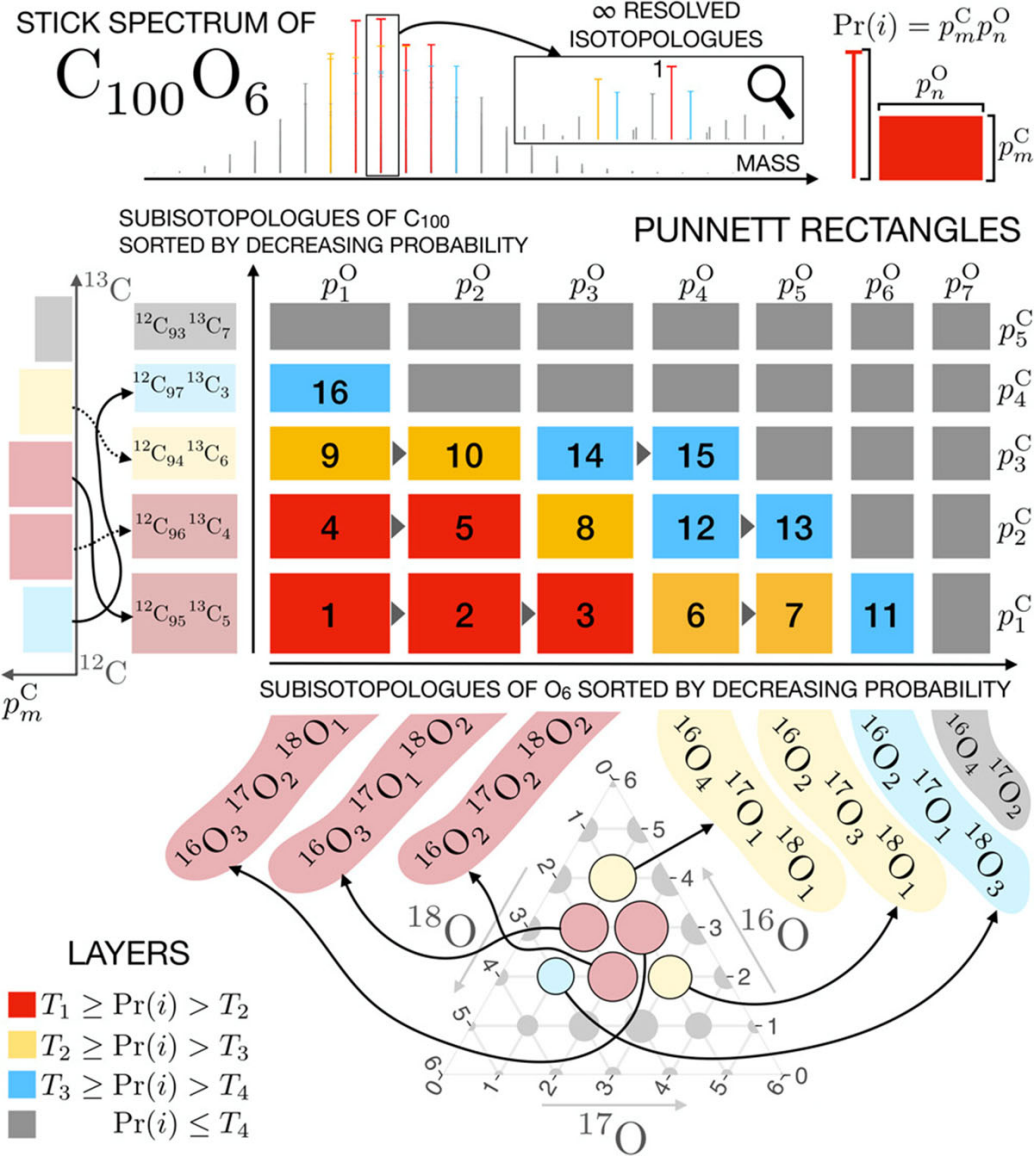


An overview of the IsoSpec algorithm: The problem is to generate the most probable mass peaks with their intensities (top). In order to do this, we represent the problem as finding a subset of the smallest size with a given probability in a space of sorted sub‐molecules consisting of isotopologues of a given chemical element. Finding the solution can be done in stages. Source: IsoSpec2: Ultrafast Fine Structure Calculator, Łącki MK. et al. *Anal Chem*. 2020;92(14):9472‐9475, https://doi.org/10.1021/acs.analchem.0c00959.

## What was your motivation for choosing postdoctoral training?

I did know the field and did not want to switch to another job after doing Ph.D. I also wanted to fix the errors I made. Obviously, by a stroke of luck, I found a new job after moving to Mainz with my wife.

## What was your biggest (if any) culture shock experience in the country of your postdoc?

It will never cease to amaze me that people in Germany knock on the tables to express they liked the talk. Obviously, clapping hands is in no way better, but where I come from people knock on things when they hope for something not to happen, in this case likely ever again.

## In your scientific career, what was the best or worst advice you ever heard from anyone?

Well, I am pretty stubborn and do not look so much for other people's advice, so I might have neglected both of those.

## What advice would you give to someone looking for a postdoc position now?

Well, weigh all your available possibilities and maybe test out different places. A scientific career seems rewarding, but it is a hard piece of bread to chew. Contrary to what may seem, academia is hypercompetitive. It is published or perish and open positions are scarce. It is a game of both luck and hard work. Want stability? Look elsewhere. Want to do cool things: Yes, you can. I am sure that though it is not the only place to do those.

## What is your favourite non‐scientific activity?

Playing with my 1‐year‐old girl and trying to teach her how to walk.

## Who (three people but not scientists!) would you invite to a dream dinner party?

The ghosts of the past, current and future Christmas.

## CONFLICT OF INTEREST

The author declares that there is no conflict of interest that could be perceived as prejudicing the impartiality of the research reported.

